# Circulating Angiopoietins-1 and -2, Angiopoietin Receptor Tie-2 and Vascular Endothelial Growth Factor-A as Biomarkers of Acute Myocardial Infarction: a Prospective Nested Case-Control Study

**DOI:** 10.1186/1471-2261-11-31

**Published:** 2011-06-14

**Authors:** Carlos Iribarren, Bruce H Phelps, Jeanne A Darbinian, Edward R McCluskey, Charles P Quesenberry, Evangelos Hytopoulos, Joseph H Vogelman, Norman Orentreich

**Affiliations:** 1 Kaiser Permanente Division of Research, 2000 Broadway, Oakland, CA 94612, USA; 2 Aviir, Inc., 2463 Faber Place, Palo Alto, CA 94303, USA; 3 Orentreich Foundation for the Advancement of Science, Inc., 855 Route 301, Cold Spring-on-Hudson, NY 10516, USA

**Keywords:** angiogenesis, acute myocardial infarction, epidemiology

## Abstract

**Background:**

Angiogenesis is up-regulated in myocardial ischemia. However, limited data exist assessing the value of circulating angiogenic biomarkers in predicting future incidence of acute myocardial infarction (AMI). Our aim was to examine the association between circulating levels of markers of angiogenesis with risk of incident acute myocardial infarction (AMI) in men and women.

**Methods:**

We performed a case-control study (nested within a large cohort of persons receiving care within Kaiser Permanente of Northern California) including 695 AMI cases and 690 controls individually matched on age, gender and race/ethnicity.

**Results:**

Median [inter-quartile range] serum concentrations of vascular endothelial growth factor-A (VEGF-A; 260 [252] vs. 235 [224] pg/mL; p = 0.01) and angiopoietin-2 (Ang-2; 1.18 [0.66] vs. 1.05 [0.58] ng/mL; p < 0.0001) were significantly higher in AMI cases than in controls. By contrast, endothelium-specific receptor tyrosine kinase (Tie-2; 14.2 [3.7] vs. 14.0 [3.1] ng/mL; p = 0.07) and angiopoietin-1 levels (Ang-1; 33.1 [13.6] vs. 32.5 [12.7] ng/mL; p = 0.52) did not differ significantly by case-control status. After adjustment for educational attainment, hypertension, diabetes, smoking, alcohol consumption, body mass index, LDL-C, HDL-C, triglycerides and C-reactive protein, each increment of 1 unit of Ang-2 as a Z score was associated with 1.17-fold (95 percent confidence interval, 1.02 to 1.35) increased odds of AMI, and the upper quartile of Ang-2, relative to the lowest quartile, was associated with 1.63-fold (95 percent confidence interval, 1.09 to 2.45) increased odds of AMI.

**Conclusions:**

Our data support a role of Ang-2 as a biomarker of incident AMI independent of traditional risk factors.

## Background

Angiogenesis, the induction and growth of new blood vessels from pre-existing ones, is a complex, highly regulated system essential for embryonic development, normal physiological growth, wound healing and tumor progression [1,2]. In addition, it is now well-documented that angiogenic factors are up-regulated (as a compensatory mechanism to increase collateral circulation) in the context of acute skeletal muscle [3] and myocardial ischemia [4-6]. Atherosclerotic vessels often present intra-plaque angiogenesis, a phenomenon that has been hypothesized to contribute to progression and eventual rupture of coronary artery lesions [7,8]. It is therefore plausible that angiogenesis could exert both protective and deleterious roles in development of coronary disease.

Vascular Endothelial Growth Factor-A (or simply VEGF), the most extensively studied angiogenic factor, is a heparin-binding homodimeric glycoprotein that induces early endothelial cell migration, proliferation and blood vessel formation [9,10]. Increased VEGF mRNA and protein expression has been demonstrated in ischemic human myocardium,^5 ^and plasma VEGF levels have been shown to be increased in patients who suffered an acute coronary syndrome (ACS) [11]. Moreover, VEGF was found to be a predictor of worse outcome after ACS [12] and was, in the Pawtucket Heart Health Program, a significant and independent predictive factor of coronary heart disease (CHD) death [13]. More recently discovered regulators of angiogenesis are the angiopoietins, which are the ligands of the endothelium-specific receptor tyrosine kinase (Tie-2) [14,15]. Whereas angiopoetin-2 (Ang-2) destabilizes the vessel to make it responsive to angiogenic growth factors such as VEGF (i.e., functions as a 'trigger' of remodeling) [16,17], angiopoietin-1 (Ang-1) promotes vascular stabilization and counteracts VEGF-induced angiogenesis [18]. In epidemiological studies, raised VEGF, Ang-2, Tie-2 but not Ang-1 have been demonstrated in patients after ACS.^11 ^In the only prior prospective study, Ang-2 but not Ang-1 was related to subsequent CVD events in a sample of 251 hypertensive patients [19].

To date, no study has examined the value of VEGF and the Angiopoietins/Tie-2 system as biomarkers of acute myocardial infarction (AMI) risk in a large population-based sample with measurement of these biomarkers in blood samples obtained before onset of AMI. Our aim was therefore to: 1) examine the prospective associations between circulating levels of VEGF, Ang-1, Ang-2 and Tie-2 with risk of incident AMI in men and women; 2) assess the degree of independence from traditional risk factors; and 3) test for, given the biological synergistic and antagonistic interrelations between these biomarkers, the six possible 2-way interactions, namely VEGF X Ang-1, VEGF X Ang-2, VEGF X Tie-2, Ang-1 X Ang-2, Ang-1 X Tie-2 and Ang-2 X Tie-2.

## Methods

### Study Population and Design

We used a nested matched case-control study design. The source population was a well-characterized, population-based cohort of 83,772 persons receiving care within Kaiser Permanente of Northern California, who participated in the Multiphasic Health Checkup (MHC) in the Oakland and San Francisco medical centers between 1984 and 1992. The MHC consisted of an innovative system of regular and comprehensive, broad-spectrum physical examinations, including routine blood testing and urinalysis, with the goal of providing health maintenance through early diagnosis [13,20-22]. Examinees were instructed not to have any food, coffee, tea or alcohol for at least four hours before the MHC appointment. At the exam, several tubes of blood were drawn and the blood was allowed to clot and then was centrifuged to separate the serum (liquid) portion from the blood cell portion. A 2 mL sample of the serum was taken and labeled, showing the sample's identification number, donor's sex, date of birth, and date of blood sampling. From this large cohort, we selected 695 incident AMI cases and we implemented incidence density sampling [23] to select 1:1 (n = 695) age-, gender-, and race-matched controls who remained free of AMI up to the date when the paired subject suffered the AMI. The incident density sampling scheme resulted in 5 controls being selected twice (stated differently, 5 AMI cases shared their respective controls with 5 other AMI cases).

Kaiser Permanente of Northern California is a large, integrated healthcare delivery system that provides care for about one-third of the insured adult population in the greater San Francisco Bay Area. Kaiser members are representative of the demographic and clinical characteristics of the surrounding local and state population with the exception of the low and high segments of the income distribution [24]. The AMI cases were identified from electronic computerized hospitalization records during the first 10 years of follow up, with the requirement that AMI (ICD-9 code 410) be listed as principal discharge diagnosis or underlying cause death and were required to have no history of prior AMI. The strategy for inclusion of events included all AMIs that occurred in the first 5 years (n = 416) plus a random selection of 279 AMI that occurred between 5 and 10 years after the MHC. This ascertainment method has been shown to have 96% specificity based on modified World Health Organization criteria of at least 2 of 3 present of: (a) ischemic symptoms, (b) ECG changes, or (c) enzyme or pathological evidence of infarction, using chart review and laboratory data as "gold standard" data sources in a prior Kaiser Permanente study [25]. The mean ± SD time between MHC and AMI was 5.2 ± 2.9 years (minimum, < 1 year; maximum, 10 years). The Kaiser Foundation Institutional Review Board approved the study protocol. VEGF and Ang-2 were measured in all AMI cases and controls. For budgetary constraints, Ang-1 and Tie-2 were measured in a 50% random sample of AMI cases and controls (in 318 case-control pairs).

### Laboratory Methods

At the MHC, serum samples were packed in dry ice in boxes of 100 and shipped overnight to the Orentreich Foundation for the Advancement of Science (OFAS), Inc., Cold Spring-on-Hudson, N.Y., where they have been computer-cataloged and maintained at -40 degrees Celsius. Aliquots of serum from the study subjects were retrieved, prepared and labeled by OFAS and shipped overnight in dry ice to Aviir, Inc., whose personnel remained blinded as to case-control status. A second aliquot was given to the Advanced Clinical Laboratory at OFAS for lipid and C-reactive protein (CRP) testing. Concentrations of VEGF and Ang-2 were measured using a LUMINEX xMAP™ Multiplex Assay, custom developed by R&D Systems (Minneapolis, MN) to measure the concentrations of 16 low abundant serum proteins. The limit of detection (LOD) for VEGF was 6 pg/ml, with an intra-assay coefficient of variation (CV) of 6% and an inter-assay CV of 8%. The LOD for Ang-2 was 6 pg/ml, with an intra-assay CV of 6% and an inter-assay CV of 7%. Concentrations of Ang-1 and Tie-2 were measured using Quantikine ELISA reagent kits and controls for each protein biomarker from R&D Systems (Minneapolis, MN, Cat # DANG10 and DTE200, respectively). HDL-C, LDL-C and triglycerides were measured on the Beckman Instruments Inc. (Brea, CA) Synchron Clinical System CX5. The CV of these measurements were less than 4.5% for HDL-C and triglycerides and less than 3% for LDL-C. CRP was measured on Siemens Health Care Diagnostics (Deerfield. IL) Immulite 1000, and the CV was less than 7%.

### Covariates

Race\ethnicity was classified as white, black, Hispanic/Latino, Asian and other by self-report. Education level was categorized as high school graduate or less, some college, and college graduate or higher. Cigarette smoking status was classified as never, former, and current. Subjects were classified according to alcohol intake (in the past year) as abstainers and consumers of < 1 drink per day (i.e., occasional), 1-2 drinks per day or 3 or more drinks per day. Height and weight were measured using standardized methods and body mass index estimated as weight in kg divided by height in m^2^. Systolic and diastolic blood pressures (one reading) were obtained in supine position with a standard manual cuff. Hypertension was defined as systolic blood pressure ≥ 140 mmHg and diastolic blood pressure ≥ 90 mmHg or use of antihypertensive medication. Diabetes mellitus was defined as self-report of a physician diagnosis or use of diabetes medications (insulin or oral hypoglycemic agents). We estimated the 10-year Framingham Risk Score for each subject as originally described [26].

### Statistical Analysis

Case-control differences were ascertained using the t-test for continuous (normally distributed) variables and the Chi-Square test for categorical variables. Because of skewness, univariate case-control differences for triglycerides, CRP and the 4 angiogenic factors were ascertained using the Wilcoxon two-sample test. To assess independent correlates of each angiogenic factors, we employed multiple linear regression with forward entry of covariates (p-stay in the model = 0.05) and forcing in a categorical variable for case-control status. Conditional logistic regression was then applied to quantify the relative odds of being an AMI case associated with 1 SD linear increment in each of the angiogenic factors. To allow comparability of effects, each angiogenic factor was log10-transformed and then scaled as a Z score by subtracting its mean and dividing by its standard deviation (SD); both means and SDs were estimated among controls. Two sequential models were fitted: first, a model without any covariates (age, sex and race adjusted for by design), and second, a model adjusting for education attainment, alcohol consumption, cigarette smoking, diabetes, hypertension, body mass index, LDL-C, HDL-C, triglycerides and CRP. We formally tested, in separate minimally-adjusted models, for all possible 2-way interactions (n = 6) between angiogenic factors as continuous variables. Because of prior research demonstrating that Ang-2 was predictive of CVD events in patients with hypertension [19], we also ran unconditional logistic models stratifying by hypertension status and formally tested the interaction between Ang-2 as a continuous variable and hypertension in both minimally-and fully-adjusted models. Finally, we ascertained strength of association as a function of time by fitting two separate conditional logistic models, one for AMI cases that occurred in the first 5 years post MHC and a second for AMI cases in years 6 to 10 post MHC. The SAS software, version 9 (SAS Institute, Cary, NC), was used in all statistical analyses.

## Results

**Figure 1:**
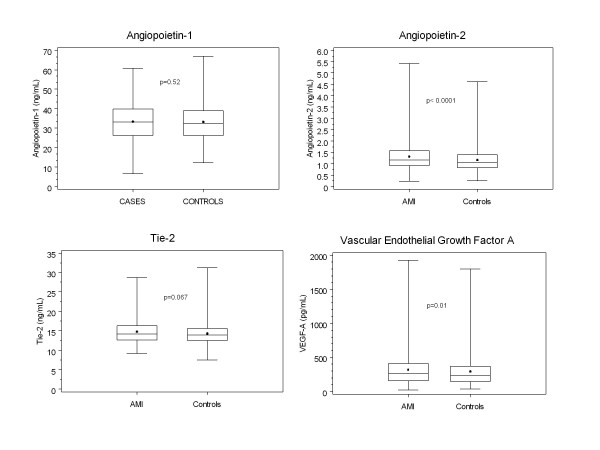
**Box plots of Angiopoietin-1, Angiopoietin-2, Tie-2, and Vascular Endothelial Growth Factor-A, by Case-Control Status showing minimum, 25th, 50th (horizontal bar), mean (dot), 75th percentile and maximum**.

Both AMI cases and controls were, on average, 62 years old at the MHC (Table 1); about 38 percent in each group were female, 54 percent white, 34 percent black, 6 percent Hispanic/Latino and 6 percent Asian. Educational attainment was lower in cases than in controls and current smoking was more prevalent in cases than in controls. Relative to controls, AMI cases tended to report less consumption of 1-2 and of 3 or more alcoholic drinks per day. As expected, a positive history of hypertension and diabetes was more frequent among cases. Mean or median BMI, triglycerides, LDL-C and CRP were higher whereas HDL-C levels were lower in cases than in controls. Based on the 10-year FRS risk calculation, 38 percent of cases and 31 of controls were at intermediate risk, whereas 30 percent of cases and 11 percent of controls were at high risk. Levels and distribution of characteristics (and the case-control patterns) were similar in the 50 percent random sample where Ang-1 and Tie-2 were measured (data not shown). Concentrations of VEGF and Ang-2 were significantly higher in AMI cases compared to controls, but no significant differences existed for Ang-1 and Tie-2 (Figure 1).

**Table 1:** Baseline characteristics of AMI cases and controls, Kaiser Permanente Multiphasic Health Checkup, 1984-1992.

	AMI Cases (n = 695)	Controls (n = 690)	p-value
	**N (%) or Mean ± SD**		

Characteristics			

Age, years*	62 ± 11	62 ± 11	

Gender*			

Men	432 (62.2)	430 (62.3)	

Women	263 (37.8)	260 (37.7)	

Race/ethnicity*			

White	377 (54.2)	373 (54.1)	

Black	235 (33.8)	234 (33.9)	

Asian	39 (5.6)	39 (5.7)	

Latino	41 (5.9)	41 (5.9)	

Other	3 (0.4)	3 (0.4)	

Education level			< 0.0001

High school graduate or less	343 (49.4)	268 (38.8)	

Some college	202 (29.1)	198 (28.7)	

College graduate or higher	150 (21.6)	224 (32.5)	

Cigarette smoking status			< 0.0001

Never	289 (41.6)	336 (48.7)	

Former	200 (28.8)	218 (31.6)	

Current	206 (29.6)	136 (19.7)	

Alcohol consumption			< 0.0001

Abstainer	79 (11.4)	45 (6.5)	

Occasional	365 (52.5)	348 (50.4)	

1-2 drinks/day	205 (29.5)	239 (34.6)	

≥ 3 drinks/day	46 (6.6)	58 (8.4)	

Hypertension	531 (76.4)	417 (60.4)	< 0.0001

Diabetes	147 (21.2)	49 (7.1)	< 0.0001

Body mass index, Kg/m^2^	27.4 ± 4.9	26.6 ± 4.7	0.002

Serum Triglycerides (mmol/L)†	1.80 (1.33)	1.38 (1.08)	< 0.0001

Serum HDL Cholesterol (mmol/L)	1.11 ± 0.34	1.27 ± 0.39	< 0.0001

Serum LDL Cholesterol (mmol/L)	2.90 ± 0.75	2.59 ± 0.72	< 0.0001

C-reactive protein (mg/L) †	2.52 (4.37)	1.55 (2.80)	< 0.0001

			

10-year Framingham Risk Score			< 0.0001

Low (< 10%)	219 (31.5)	399 (57.8)	

Intermediate (10- < 20%)	265 (38.1)	212 (30.7)	

High (≥ 20%)	211 (30.4)	79 (11.4)	

			

Angiopoietin-1 (ng/ml) †‡	33.1 (13.6)	32.5 (12.7)	0.52

Angiopoietin-2 (ng/ml) †	1.18 (0.66)	1.05 (0.58)	< 0.0001

Tie-2 (ng/ml) †‡	14.2 (3.7)	14.0 (3.1)	0.07

Vascular Endothelial Growth Factor-A (pg/ml) †	260 (252)	235 (224)	0.01

* Matching factors; † Median (Interquartile range); ‡ measured in a 50% random sample

Table 2 summarizes the bivariate correlations amongst continuous variables. The highest correlation was noted between triglycerides and HDL-C (r = -0.55). The correlations between the four angiogenic factors were weak (r = 0.14 or lower), and the correlations between CRP and angiogenic factors were also weak ranging from 0.22 for Ang-2 and 0.16 for Ang-1. The independent correlates of VEGF, accounting for 6 percent of its variance were CRP, hypertension, less than a high school education, current smoking and triglycerides (Table 3). The factors independently related to Ang-2 were CRP, diabetes, current smoking, male gender, BMI, age, consumption of 3 or more alcoholic drinks per day, LDL-C and HDL-C, accounting for 18 percent of its variance (Table 3). CRP, LDL-C, male gender and occasional alcohol consumption were the independent predictors of Ang-1, accounting for 6 percent of its variance (Table 3). In turn, the independent covariables of Tie-2 were diabetes, HDL-C, CRP and triglycerides, and these variables accounted for 12 percent of the variation in Tie-2 levels (Table 3). Results of the conditional logistic regression analysis are presented in Table 4. In the minimally adjusted model, there were significantly increased odds of AMI associated with linear increases in VEGF, Ang-2 and Tie-2, and no association with Ang-1. The strength of association was greater for Ang-2, lower for VEGF and intermediate for Tie-2. After adjustments for educational attainment, hypertension, diabetes, smoking, alcohol consumption, BMI, LDL-C, HDL-C, triglycerides and CRP, the association between VEGF and AMI was attenuated and became non-statistically significant. The association between Ang-2 and AMI was also attenuated after multivariate adjustment but remained statistically significant. A noteworthy finding was that a linear increment of Tie-2 became significantly associated with decreased odds of AMI after adjustment for all study covariates. An additional fully-adjusted model entering categorical variables for quartiles of Ang-2 (cut-points defined among controls), quartiles 2, 3 and 4, relative to quartile 1, were associated with 1.25 (95% CI, 0.85 to 1.84), 1.38 (95% CI, 0.93 to 2.04) and 1.63 (95% CI, 1.09 to 2.45) increased odds of AMI, respectively. In fully-adjusted conditional logistic models stratified by years to AMI, the OR associated with 1 SD increment of Ang-2 (as a Z score) were 1.16 (95% CI, 0.96 to 1.41) and 1.21 (95% CI, 0.97 to 1.51) during the first 5 years and during years 6 to 10, respectively.

**Table 2:** Spearman correlations* amongst continuous variables (n = 1,380).

	VEGF-A	Ang-2	**Ang-1**†	**Tie-2**†	CRP	LDL	HDL	Triglycerides	Age	BMI
**VEGF-A**	1.00	0.11	0.31	0.06	0.19	0.02	-0.06	0.09	-0.01	0.07

**Ang-2**		1.00	-0.04	0.14	0.22	-0.03	-0.05	0.07	0.09	0.19

**Ang-1**†			1.00	0.01	0.16	0.14	0.06	0.06	0.04	-0.003

**Tie-2**†				1.00	0.20	0.04	-0.19	0.16	-0.05	0.11

**CRP**					1.00	0.09	-0.17	0.20	0.02	0.38

**LDL**						1.00	0.03	0.28	0.01	0.04

**HDL**							1.00	-0.55	0.13	-0.23

**Triglycerides**								1.00	-0.001	0.25

**Age**									1.00	-0.11

**BMI**										1.00

* Controlling for case-control status; † measured in a 50% random sample (n = 634)

**Table 3:** Independent predictors of angiogenic factors.

Angiogenic Factor	β	SE	p	Model R^2^
VEGF-A (pg/mL)*				

Log CRP/SD	39.4	6.0	< 0.0001	0.040

Hypertension	30.3	13.1	< 0.0001	0.046

High school graduate or less	-30.3	14.8	0.04	0.051

Current smoking	39.8	15.1	0.008	0.057

Log triglycerides/SD	12.6	6.0	0.03	0.059

Ang-2 (ng/mL)*				

Log CRP/SD	0.061	0.016	< 0.0001	0.06

Diabetes	0.244	0.043	< 0.0001	0.09

Current smoking	0.278	0.038	< 0.0001	0.12

Male gender	-0.17	0.034	< 0.0001	0.13

BMI/SD	0.071	0.016	< 0.0001	0.15

Age/10 years	0.069	0.015	< 0.0001	0.16

Alcohol consumption ≥ 3 drinks/day	0.222	0.059	0.0002	0.17

**Ang-1 (ng/mL)†**				

Log CRP/SD	1.16	0.37	0.002	0.02

LDL/SD	1.01	0.38	0.008	0.03

Male gender	-2.14	0.83	0.009	0.04

Occasional alcohol consumption	-2.85	0.89	0.001	0.06

**Tie-2 (ng/mL)†**				

Diabetes	1.91	0.32	< 0.0001	0.07

HDL/SD	-0.48	0.12	< 0.0001	0.10

Log CRP/SD	0.36	0.11	< 0.0001	0.11

Log triglycerides/SD	0.25	0.13	0.05	0.12

* n = 1,380; controlling for case-control status

† n = 630; controlling for case-control status

**Table 4:** Unadjusted and adjusted odds ratios and 95 percent confidence intervals of acute myocardial infarction from 4 separate models.

Angiogenic factors	Minimally-adjusted*	**Adjusted**†
VEGF-A, 1 unit of Z score	1.13 (1.02-1.26)	1.01 (0.89-1.15)

Ang-2, 1 unit of Z score	1.31 (1.17-1.46)	1.17 (1.02-1.35)

Ang-1, 1 unit of Z score	0.98 (0.84-1.13)	0.95 (0.78-1.15)

Tie-2, 1 unit of Z score	1.19 (1.01-1.40)	0.79 (0.63-0.98)

* Adjusted for matching factors (age, gender, race/ethnicity) by design

† additional adjustment for diabetes, hypertension, smoking, alcohol consumption, BMI, LDL, HDL, triglycerides and CRP

using a 50% random sample

**Figure 2:**
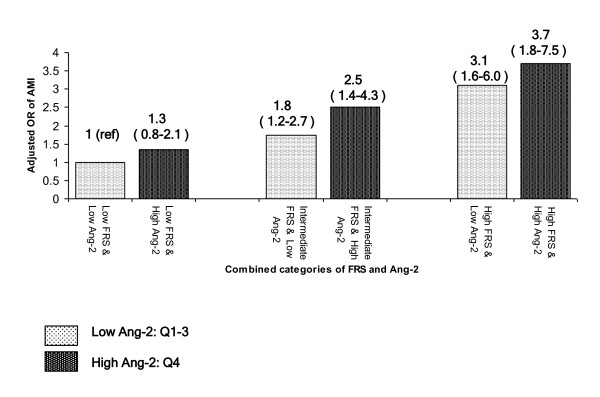
**Combined Effect of 10-year Framingham Risk and Ang-2 on Odds Ratio of AMI**. Low Ang-2 (light pattern) defined as quartiles 1 through 3; high Ang-2 (heavy pattern) defined as quartile 4.

In six separate minimally-adjusted models, each including main and interactive effects for the two angiogenic factors considered, no evidence was found of significant 2-way interactions (all p-values ≥ 0.09). When the sample of AMI cases and controls was divided into 6 groups according to low, intermediate and high 10-year Framingham risk crossed with low/high Ang-2 (defining low Ang-2 as quartiles 1 through 3 and high Ang-2 as quartile 4 and considering low FRS/low Ang-2 as referent), a consistent pattern emerged such that in each FRS category there was a gradient of increased adjusted odds of AMI moving from low to high Ang-2 (Figure 2). However, the confidence intervals overlapped in each of the three FRS levels, and thus there were no statistical significant differences in adjusted odds of AMI comparing low vs. high Ang-2 within each of the FRS categories. In minimally-adjusted unmatched logistic regression stratified by hypertension status, each increment of Ang-2 (1 unit of its Z score) was associated with 1.16 (95% CI, 0.95 to 1.42) increased odds of AMI among those without hypertension and with 1.30 (95% CI, 1.13 to 1.48) increased odds of AMI among those with hypertension. These OR estimates were 1.04 (95% CI, 0.83 to 1.31) and 1.12 (0.96 to 1.31), respectively, after full multivariate adjustment within hypertension strata. The p-values for Ang-2 by hypertension interaction were 0.47 in the minimally-adjusted model and 0.54 in the fully-adjusted model.

## Discussion

Angiogenesis is essential for the repair of wounds and tissues damaged by ischemia (and for the process whereby collateral circulation develops in the context of ischemia), but it also may enhance neovascularization-dependent disease such as tumor or atherosclerotic plaque [4-8]. In this population-based case-control study, elevated levels of Ang-2 were significantly and independently related to future likelihood of AMI, which is consistent with the notion that an angiogenic compensatory response to silent ischemia or subclinical atherosclerosis may exist even years before the clinical presentation of AMI. Two other angiogenic factors, VEGF and Tie-2 were also associated with increased future odds of AMI in minimally-adjusted analyses, but their respective association became null and protective, respectively, after multivariable adjustment for traditional risk factors and CRP.

In disagreement with our findings, a prior population-based prospective study reported that baseline levels of VEGF-A were significantly and independently associated with the risk of CHD death [13]. There are some differences between that study and ours that may explain this discrepancy in results. First the Eaton et al. study was a case-cohort design whereas ours was a case-control matched design. Second, the Eaton et al study focused on fatal CHD, while our endpoint was both non-fatal or fatal AMI. Unlike Eaton et al., we measured and adjusted for CRP in the multivariable model (CRP and VEGF correlated 0.19 in our sample). Lastly, the study sample in Eaton et al. was 92% white, while our samples of AMI cases and matched controls were 54% white.

Our null (albeit observational) results for VEGF fit with the current evidence from rigorous phase II and III clinical trials of VEGF gene therapy demonstrating no benefit [27]. A new promising paradigm for therapeutic angiogenesis is emerging that incorporates multiple growth factors and "master switch" agents [28,29].

The fact that Tie-2 had a positive association in the minimally-adjusted model that became protective in the multivariate context is somewhat perplexing and deserves some comment. In sensitivity analysis adding one covariate at a time, the largest attenuation and reversal of directionality of association was apparent with the inclusion of HDL-C, which was one of the strongest covariates of Tie-2. This finding warrants further research in other populations and locations.

Our findings also suggest that the predictive ability of Ang-2 did not vary with years to event (similar effects were seen regardless of whether the AMI had occurred in the first 5 years of follow-up or in the subsequent 6-10 years of follow-up). We found a trend toward a stronger Ang-2-AMI association among those with hypertension (compared to those without hypertension) but failed to demonstrate a significant interaction between these two factors. Our results among hypertensive participants are in agreement with a study in the UK concluding that raised levels of Ang-2 were predictive of AMI among 251 patients with hypertension [19].

The strengths of our study include the availability of serum samples many years before the development of AMI in case subjects. Another strength is the ethnic diversity of the AMI cases and controls. Our study has a number of limitations that should be appreciated. First, a legitimate concern is protein degradation as a result of the advanced age and conditions of storage of the serum samples. This is not likely to have biased our results since the mean (SD) concentrations of VEGF in our controls (295 [235] pg/ml) is similar to the mean (SD) levels reported by Eaton et al. for the entire sub-cohort (303 [219] pg/ml) [13]. In addition, to specifically address the integrity of the serum bank, OFAS measured the level of dehydroepiandrosterone sulfate (DHEAS), a common hormone that declines over the life span, in 517 of these long-frozen samples from women in 13 different age groups. Levels of DHEAS in the 517 long-frozen sera correlated extremely well with levels of DHEAS in the 2,500 unfrozen clinical samples, and this excellent correlation established that the chemical integrity of the serum had been maintained over the years. Additional protein studies using this serum bank have also confirmed the chemical integrity of the samples [28]. Moreover, to determine if desiccation had occurred, the serum concentration of sodium was measured in each specimen with a Beckman model E2A analyzer (Irvine, CA) and none of our samples were outside the physiological normal range of 135-153 mmol/L. Another limitation of our study is that we did not have measures of other angiogenic factors such as bFGF (basic fibroblast growth factor) [30], endostatin [31] or angiostatin [32], thought to play important roles in collateral vessel formation and plaque angiogenesis. A third (methodological) limitation is that our matched design precluded estimation of incremental discrimination by ROC analysis or the net reclassification improvement [33] after considering Ang-2. This is because estimation of association is via conditional logistic regression, appropriate to the nested case-control study design, which does not provide estimates of the intercepts. Finally, we did not have information on medical or surgical management of the AMI cases, although the likelihood of these interventions affecting the measurement of angiogenic factors is very low because the samples utilized antedated the event up to 10 years and were obtained when the patients were asymptomatic.

## Conclusions

This study provides further evidence supporting a role of biomarkers of angiogenesis in the prediction of AMI. In particular, Ang-2 emerged as a particularly promising biomarker. These findings should motivate additional research to elucidate the usefulness of Ang-2 in CVD risk stratification either alone or as part of multiple biomarker approaches.

## Competing interests

Carlos Iribarren received a research grant > 10K from Aviir, Inc. Bruce H. Phelps is an Aviir, Inc. employee (CTO) and holds Aviir, Inc. stock options. Jeanne A. Darbinian has no conflicts to disclose. Edward R. McCluskey is an Aviir, Inc. employee and Aviir, Inc. and holds Aviir, Inc. stock options. Charles P. Quesenberry has no conflicts to disclose. Evangelos Hytopoulos is an Aviir, Inc. employee and holds Aviir, Inc. stock options. Joseph H. Vogelman has no conflicts to disclose.

Norman Orentreich has no conflicts to disclose.

## Authors' contributions

All authors have read and approved the final manuscript. CI obtained the funding for the study, drafted and revised the article, and supervised the statistical analysis. BHP supervised assay performance and provided a critical review of the article. JAD performed the statistical analysis and provided a critical review of the article. ERM provided a critical review of the article. CPQ gave statistical advise and provided a critical review of the article. EH gave statistical advise and provided a critical review of the article. JHV supervised the sample identification, retrieval and shipping, suipervised the lipid and CRP assays and provided a critical review of the article. NO provided a critical review of the article.

## Abbreviations

AMI: Acute myocardial infarction; ACS: Acute coronary syndrome; FRS: Framingham Risk Score; OR: Odds ratio; 95% CI: 95 percent confidence intervals; MHC: Multiphasic health checkup; OFAS: Orentreich Foundation for the Advancement of Science; SD: Standard deviation; LDL: Low density lipoprotein; ANG-1: Angiopoietin-1; ANG-2: Angiopoietin-2; VEGF: Vascular endothelial growth factor; VEGF-A: Vascular endothelial growth factor-A; Tie2: Endothelium-specific receptor tyrosine kinase

## Pre-publication history

The pre-publication history for this paper can be accessed here:

http://www.biomedcentral.com/1471-2261/11/31/prepub
